# The role of the pediatrician in the management of the child and adolescent with gender dysphoria

**DOI:** 10.1186/s13052-023-01466-z

**Published:** 2023-06-14

**Authors:** Ginevra Micangeli, Giovanni Profeta, Fiorenza Colloridi, Federica Pirro, Francesca Tarani, Giampiero Ferraguti, Matteo Spaziani, Andrea M. Isidori, Michela Menghi, Marco Fiore, Luigi Tarani

**Affiliations:** 1 Department of Pediatrics, “Sapienza” University of Rome, Rome, Italy; 2 Department of Experimental Medicine, Sapienza University of Rome, Rome, Italy; 3 Department of Experimental Medicine, Section of Medical Pathophysiology, Food Science and Endocrinology, Sapienza University of Rome, Rome, Italy; 4 Institute of Biochemistry and Cell Biology, IBBC-CNR, Rome, Italy

**Keywords:** Gender dysphoria, Pediatrician, Gender affirming surgery, Assigned female at birth, Hormone therapy, Multidisciplinary approach, Reversibility

## Abstract

Gender dysphoria is a clinical condition characterized by significant distress due to the discordance between biological sex and gender identity. Currently, gender dysphoria is also found more frequently in children and adolescents, thanks to greater social sensibleness and new therapeutic possibilities. In fact, it is estimated that the prevalence of gender dysphoria in pediatric age is between 0.5% and 2% based on the statistics of the various countries. Therefore, the pediatrician cannot fail to update himself on these issues and above all should be the reference figure in the management of these patients. Even if the patient must be directed to a referral center and be followed up by a multidisciplinary team, the treating pediatrician will care to coordinate the clinical and therapeutic framework. The aim of the present report is therefore to integrate literature data with our clinical experience to propose a new clinical approach in which the pediatrician should be the reference in the care of these patients, directing them towards the best therapeutic approach and staying in contact with the specialists of the referral center.

**Supplementary Information:**

The online version contains supplementary material available at 10.1186/s13052-023-01466-z.

## Introduction

**Gender dysphoria (GD)** is a condition characterized by significant distress due to discordance between biological sex and gender identity [1, 2]. However, to better understand what has just been stated, it is important to clarify the terminology that concerns **sexual identity**, which can be described as the subjective perception of the individual’s sexuality consisting of the following components [3, 4]. The **biological sex** is genetically defined by the sex chromosomes and genes which determine the gonadal sex that, through the sex hormones will determine the phenotypic sex [4]. Therefore, it has specific anatomical characteristics that allow distinguishing the female from the male. There are more complex cases in which due to sexual development disorders (DSD) these characteristics are not completely defined, named also with the abandoned term **intersex** [5, 6].

**Gender identity** instead, is the feeling of being male or female that everyone has. This is a psychological identity that is acquired during growth. Indeed, children consolidate their gender identity towards 3–4 years of age [7, 8]. **Gender role** can be defined as the set of behaviors and roles on how males and females should behave [9]. These are usually related to cultural patterns and influenced by the historical period: by these means, girls are expected to play with dolls and use skirts and boys prefer football and contact sports. It is important to keep in mind that one may not conform to these cultural constructs without altering the perception of belonging to one’s gender [10].

At the same time, we must speak of **sexual orientation** defined as the physical, emotional and erotic attraction that one feels towards another person [3, 11]. Three categories are commonly defined: heterosexuality if you are attracted to people of the opposite sex, homosexuality if you are attracted to people of the same sex and bisexuality if you are attracted to both sexes [12]. In reality, according to the latest sociological models, it would be more correct to speak of the **sexual spectrum** rather than sexual orientation, meaning it as something fluid and dynamic, which can be changed over time in its individual nuances without having to fall within the rigid labels mentioned above [13, 14].

And last but not least, we have to define the meaning of *i)***cisgender** which describes a person whose gender identity aligns in a traditional sense with the sex assigned to them at birth and the meaning of *ii)***transgender**, an umbrella term describing individuals whose gender identity does not align in a traditional sense with the gender they were assigned at birth [9, 15]. It may also be used to refer to a person whose gender identity is non-binary and not traditionally associated with that assigned at birth [16–19].

The term transgender, therefore, includes the following definitions:

- **Genderqueer** is a person with a non-binary identity. Many genderqueer people do not recognize themselves in the gender assigned to them at birth and also define themselves as transgender [20].
- **Genderfluid** represents a gender identity that fluctuates along the gender spectrum, varying over time. A genderfluid person can at any time identify himself as male, female, neutral or any other non-binary identity.
- **Agender or Genderless**: It means having a non-binary gender identity or not having a gender identity.
- **Bigender**: which has the meaning of experiencing both gender identities in detail, this can happen simultaneously or separately [21].
- Transsexual: a person who has faced or is facing a gender affirmation path through the assumption of hormones and medical-surgical interventions aimed at reaffirming the physical-psychological identity, through the modification of the sexual organs [22].

This terminology should not be associated with that of transvestism. Indeed, the transvestism is a form of fetishism, the clothes are the fetish, which in turn is a type of paraphilia [23]. In transvestism, the man prefers to wear women’s clothing or, less frequently, the woman chooses to wear men’s clothing. However, cross-dressers do not necessarily have an inner sense of belonging to the opposite sex or even the desire to change gender, as occurs in gender dysphoria [8, 24].

It is important, given the complexity of the subject, to use terminology correctly in order not to create confusion. In most individuals, the gender identity corresponds to the biological sex, thus identifying itself in one of the two different extremes, the male and the female. Some subjects, however, don’t find a precise place in one of the two genders and fall into what is defined as **gender non-conformity** or **gender variance** [10, 25].

**Gender variance (GV)** is not to be considered pathologic, in fact, children consolidate their gender identity around the age of three, while around the age of five most of them need to apply the expected behavior based on their gender [25, 26]. At the same time, some children may show gender non-conformity, preferring games or clothes that do not coincide with what is culturally expected. For example, there may be girls who prefer to play football or boys who prefer to dance, but this doesn’t mean that the children must identify with another gender than their biological sex [27, 28] It is therefore evident that for many children the experience of gender non-conformity is transitory, resolving at puberty. At the same time, when the individual experiences strong emotional, psychological and physical discomfort due to the incongruence between the experienced gender and the biological sex it is referred to as **GD** [1, 29].

To make a diagnosis of gender dysphoria it is necessary to follow the diagnostic criteria of the DSM-V of 2013 [2]. These have eliminated the previous definition of *gender identity disorders* replacing it with the one of *gender dysphoria* and have also provided for a series of exclusive criteria for diagnosis in children, thus creating a separate classification compared to adolescents and adults [2, 30, 31]. This condition is still considered by the DSM-5 as a mental disorder, while the ICD-11 has redefined gender identity-related health, replacing outdated diagnostic categories like ICD-10’s “transsexualism” and “gender identity disorder of children” with “gender incongruence of adolescence and adulthood” and “gender incongruence of childhood”, respectively. Gender incongruence has been moved out of the “Mental and behavioral disorders” chapter and into the new “Conditions related to sexual health” chapter [32, 33]. This reflects current knowledge that trans-related and gender diverse identities are not conditions of mental ill-health, and that classifying them as such can cause enormous stigma [34]. It remains in the diagnostic manual as there are still relevant care and health needs that can be better satisfied, according to the authors of the manual, if this condition continues to be codified in the diagnostic manual itself [23, 35]. Actually, the issue is complicated because on the one hand, thinking of depathologizing gender dysphoria and removing it from the DSM-V would reduce the social stigma that still accompanies this condition [34, 36]. On the other hand, recognizing it as a pathology allows to draw up a clear diagnostic-therapeutic procedure and facilitate access to treatment for the people concerned. The diagnostic criteria of DSM-V for children and adolescents or adults are those in Tables 1 and 2.

**Table 1:** DSM-V criteria for gender dysphoria in children [2, 83]. The table lists the current diagnostic criteria proposed by the 2013 DSM-V for the diagnosis of gender dysphoria in children

A marked incongruence between one’s experienced/expressed gender and assigned gender, of at least 6 months duration, as manifested by at least 6 of the following indicators: • A strong desire to be of the other gender or an insistence that he or she is the other gender. • In boys, a strong preference for cross-dressing or simulating female attire; in girls, a strong preference for wearing only typical masculine clothing and a strong resistance to the wearing of typical feminine clothing. • A strong preference for cross-gender roles in make-believe or fantasy play. • A strong preference for the toys, games, or activities typical of the other Gender. • A strong preference for playmates of the other gender. • In boys, a strong rejection of typically masculine toys, games, and activities and a strong avoidance of rough-and-tumble play; in girls, a strong rejection of typical feminine toys, games, and activities. • A strong dislike of one’s sexual anatomy. • A strong desire for the primary and/or secondary sex characteristics that match one’s experienced gender. In order to meet criteria for the diagnosis, the condition must also be associated with clinically significant distress or impairment in social, school, or other important areas of functioning [83].	

**Table 2:** DSM-V criteria for gender dysphoria in adolescents and adults [2, 30, 31]. The table lists the current diagnostic criteria proposed by the 2013 DSM-V for the diagnosis of gender dysphoria in adolescents and adults

A marked incongruence between one’s experienced/expressed gender and their assigned gender, lasting at least 6 months, as manifested by at least two of the following: • A marked incongruence between one’s experienced/expressed gender and primary and/or secondary sex characteristics (or in young adolescents, the anticipated secondary sex characteristics) • A strong desire to be rid of one’s primary and/or secondary sex characteristics because of a marked incongruence with one’s experienced/expressed gender (or in young adolescents, a desire to prevent the development of the anticipated secondary sex characteristics) • A strong desire for the primary and/or secondary sex characteristics of the other gender • A strong desire to be of the other gender (or some alternative gender different from one’s assigned gender) • A strong desire to be treated as the other gender (or some alternative gender different from one’s assigned gender) • A strong conviction that one has the typical feelings and reactions of the other gender (or some alternative gender different from one’s assigned gender) In order to meet criteria for the diagnosis, the condition must also be associated with clinically significant distress or impairment in social, occupational, or other important areas of functioning [87].	

The aim of this narrative review is to improve the clinical management of patients with gender dysphoria by integrating information from the scientific literature (PubMed) with that of our clinical experience. In fact, we do believe that a *multidisciplinary* approach integrated with other specialists is essential to favor the correct clinical therapeutic pathway for these patients.

## Epidemiology

Due to the lack of epidemiological studies in the literature, there are no reliable and current data on the real case history. Certainly, although dysphoria can be considered a rare condition, there has been an increase in the prevalence of gender dysphoria and it’s not clear whether this is attributable to a greater coming out due to higher social tolerance and possibilities for intervention and therapeutics or if it is really correlated to an increase in the number of cases [19, 37].

It should be noted there is also a variability according to the various nations, this is probably due to cultural and social models [38]. For example, in a 2013 California study on a sample of 2730 primary school children, 1.3% identified themselves as transgender when asked “What gender do you belong to?“ [12]. In a New Zealand study of 8166 high school students, 1.2% of them answered positively to the question “Do you think you are transgender?“ [39] In another study conducted in Minnesota in 2016 on 81,855 high school students, 3.6% of girls and 1.7% of boys answered yes to the following question “Do you consider yourself transgender, genderqueer, genderfluid or unsure about your gender identity?“ [40]. What emerged from the few available studies and the specific questionnaires is that in children there is a higher prevalence in males than in females, with a ratio that varies from 3:1 to 6:1 according to the various case series. On the other hand, in adolescents, the ratio would seem to be 1:1 [41, 42].

Compared to the prevalence of GD in adolescence (and in adulthood), studies based on clinical samples indicate that GD is relatively rare (approximately 1:7,400; 1:100,000 in male births and 1: 30.400; 1:400.000 in girls born) [43]. Even in this case, these numbers are likely underestimated since most of the studies are based on visits to GD specialized services and do not consider individuals experiencing mild GD and/or who do not apply for medical interventions.

As for adults is estimated a prevalence of about 5–14 cases per 1000 inhabitants in men and 2–3 cases per 1000 inhabitants in women, making it a much more common condition than has emerged in previous decades [19, 38, 44] If we refer to the gender variance, the numbers are even higher for example in an Italian study conducted on 350 children aged between 3 and 5 years, 5.2% of males and 3.9% of females showed a gender variance following the administration of the *Gender identity interview for the child* [7]. From a report by the Italian National Observatory of Gender Identity (ONIG) it is estimated that from 2005 to 2018 in the 8 national reference centers for minors, about 251 patients were taken in care with a progressive increase over the years, confirming the fact that in Italy the numbers are lower than in the rest of Europe. In Italy it has been estimated a prevalence of 1:12,000 males who want to become females and 1:30,000 females who want to become males [45]. Although these data are official even according to the same organization, they underestimate the real prevalence of the GD which should fluctuate between 0.5 and 2% in the pediatric age [46].

## Etiology

Although there are no definitive etiopathological hypotheses on the development of gender dysphoria, there is general agreement on the fact that it recognizes a multifactorial etiopathogenesis. It is therefore considered as a clinical picture where factors of different types intervene: biological, social, cultural and psychological [47, 48].

### The genetic hypothesis

As for the genetic aspect, numerous genes have been studied regarding gender identity, assuming that sex hormones are the basis of sexual differentiation in the brain. For instance, it was found that there are no associations with any polymorphisms of the gene coding for 5-alpha-reductase in both male and female transgender subjects [47, 49]. Relevant data were obtained from studies conducted on girls with complete adrenogenital hyperplasia with 46 XX karyotype, from which it emerged that girls had greater tendencies in gender variance than controls up to 40.9%, even if only in a few cases it was described as a real gender dysphoria [50]. This was higher in this group of patients with values up to 3% compared to 0.002–0.003% of the general population [50].

Certainly, the hormones involved in puberty may have a role in influencing gender identity. Studies conducted on people with 5 alpha-reductase deficiencies showed that about 50% of these patients changed gender identity during puberty [51]. On the contrary, in pathologies affecting the sex chromosomes, such as Turner syndrome (45 × 0) or Klinefelter syndrome (46 XXY), there are no increases in the rate of GD, except in a small percentage of patients with Klinefelter with an underlying autism spectrum disorder [52–55]. What emerges from the literature is in fact that patients with autism spectrum disorders of pediatric age have higher rates of GD than the general population, although the concordance of these two disorders is not fully known [56, 57].

Although several studies support the possibility of a genetic or hormonal etiopathogenesis, it is more correct to speak of multifactorial as there are no univocal and reproducible data in the literature [38, 58, 59].

### The neurobiological hypothesis

From a neurobiological point of view in the literature, there are several studies on the neuroanatomical differences between men and women and on the consequent differences in transgender individuals. Different areas of the brain were studied to evaluate the differences that could emerge between transgender and cisgender subjects. For example, in a study conducted in 2013 it emerged that in the group of transgender subjects studied, both men and women, there was a reduction of gray matter in the cerebellum and the left angular gyrus compared to cisgender controls [60]. This finding discloses a difference in the two groups in areas that are necessary for controlling the perception of one’s own body and for recognizing faces [60]. In a study conducted in 2018, it was shown that transgender people had an increased cortical thickness in the prefrontal mesial region and the left area of the occipitotemporal cortex [61]. Furthermore, a study conducted in 2015 found a reduced hemispheric connection between the subcortical and limbic areas in transgender subjects compared to those studied [62].

From the point of view of neurological mediators, neurotrophins may play key roles [63–66]. Neurotrophins are active not only on nerve cells but also play a key role as endocrine and paracrine regulators of the cardiovascular, immune and endocrine systems to regulate homeostasis in physiological and/or pathological conditions [66–68]. Indeed, the brain-derived neurotrophic factor (BDNF) is normally affected under stress conditions [69, 70] slightly increased in the serum of individuals with GD compared to the control group, even if in this study, patients with depressive diseases were not excluded which could be the underlying reason for the rise of BDNF [71]. At the same time, in a 2017 study, it was observed that patients with GD undergoing surgical treatment do not have a significant reduction in BDNF as would be expected [72].

Markers of neuroinflammation were also affected in transgender individuals because exposed to stressful events from childhood to adulthood [73, 74]. Interleukin (IL)-1β, IL-6, IL-10 and tumor necrosis factor-alpha (TNF-α) are inflammatory cytokines that regulate our immune system [75–77]. Imbalanced levels in such cytokines are linked to history of childhood maltreatment and psychiatric disorders [73].

Even today the neurobiological causes behind GD are not fully elucidated, although several possible hypotheses have been studied [78].

## The role of the pediatrician in the clinical practice

It must be considered that being a condition on the rise and no longer so rare, the pediatrician should know how to correctly act upon the management of these patients. In fact, very often the first clinician evaluating these children and adolescents is the pediatrician who has the delicate task of taking care of these patients, directing them toward the best treatment path [79–82].

The pediatrician is forced to face different ethical-clinical considerations: the GD is a real disorder or a variant of behaviors gender dependent? How important are cultural factors in the epidemiological data of the GD and what is the prevalence of the GD in cultures different from the Western ones? GD causes suffering for itself or through social and cultural issues? If a teenager asks for immediate hormonal treatment or surgery, have the doctor to agree? How and what to respond to parents who ask for corrective treatment? Adults often trivialize, they tend to minimize, but, albeit in small numbers, some of these children with GD will ask to be helped to make choices that will affect their lives in a decisive way. What the pediatrician should do in the first place is to use inclusive and non-judgmental language and help parents to have an accommodating attitude towards their children. Indeed, it is useless to intervene with coercive methods such as punishments, denigrating languages, or devaluing attitudes. What could happen with this type of approach is that the child can change his/her gender role, not modulating the gender identity which would be resistant to family interventions [83, 84].

It is essential that the pediatrician is in any case supported by the neuropsychiatrist in the correct management of the patient suffering from gender dysphoria. The neuropsychiatrist should already be present from the first evaluation of the patient with suspected dysphoria to accompany the pediatrician in the correct identification of the diagnostic criteria as established by the DSM-V [85]. Indeed, the neuropsychiatrist should always be consulted in the suspicion of a patient suffering from gender dysphoria and should always be present to communicate the diagnosis to the patient and his family, in this way he can also act as a bridge to direct the patient toward the reference center [86–88].

It has been demonstrated that patients who are managed by a multidisciplinary team have a mental outcome comparable to that of the general population [89]. One of the most important things in the management of these patients is certainly knowing how to listen and identify the problems [90]. This crucial issue is something the pediatrician should always do when dealing with complex patients [90].

One of the pediatrician crucial tasks is to sustain the patient by guaranteeing support not only at a clinical level, but above all in the social environment in which lives. Indeed, in the case of a patient with GD, it would be appropriate for the clinician to relate to the school staff, trying to make the problems connected to GD understood [35]. The meeting with the teachers must be exhaustive and must direct them to understand how to manage the patient, favoring their correct inclusion in the class group [91].

Children and adolescents with GD or GV perceive that they live as if they were in the wrong body, they feel distressed, confused, alone and share their emotions with extreme difficulty with the outside and consequently may have greater relationship difficulties than their peers. Certainly, the social stigma, isolation and psychological distress to which they are exposed make them more susceptible to substance abuse, self-harm and suicide attempts and predispose them to a greater risk of becoming victims of bullying and marginalization. This can affect their quality of life with an increased risk of psychiatric disorders, suicide and social maladjustment [92]. Interestingly, in many children the gender variance is transitory and usually with puberty it tends to resolve without causing gender dysphoria and according to some clinical studies it persisted in only 6–23% of adult subjects [79]. These children more frequently express a homosexual or bisexual orientation [3, 93].

On the other side, in adolescents the persistence of gender dysphoria has significantly higher rates than in children, usually, the persistence or desistance of GD occurs around 10–13 years of age [94]. Although there are no easily identifiable predictors to identify which children will have a persistence of GD, some have been hypothesized, including the belief and insistence of their claims about their belonging to the biological sex of birth, the severity of their dysphoria and their cross-gender behaviors during childhood [29, 84].

For this reason, the pediatrician should know how to communicate with the family, explaining that not all children who exhibit cross-gender behaviors will have GD in adulthood. In this regard, the treating pediatrician must use an appropriate terminology to explain the differences between gender dysphoria and gender variance. He should also explain to parents how cultural models can influence gender roles and how not all children respond to the behavior dictated by the society. In fact, some of them follow models, both in play and in clothing, belonging culturally to the opposite sex while having no doubts about their own gender identity.

It is clear how essential it is that the pediatrician is ready to answer all the questions that parents may ask and above all this must be able to guide the family in a path of acceptance and inclusiveness towards the child affected by GD [95]. For this reason, the doctor must be present for the family and must act as a bridge with the other social environments with which the child interacts, such as school, and any sporting or hobby activities [96].

As for the school environment, in addition to interacting with the teachers, the pediatrician should take care of organizing sexual-affective education meetings to raise awareness among his classmates [96]. These meetings should be held in conjunction with subject matter experts such as neuropsychiatrists or psychologists, and should focus on correct terminology and inclusiveness [95]. We know that today this is a hotly debated topic, especially in Italy, but we believe that proper awareness raising and information for school-age children may promote tolerance and the inclusiveness of what we call diversity.

Furthermore, the pediatrician should pay attention to the possibility of psychiatric comorbidities in these patients. In fact, what emerges from the literature is that adolescence with GD has higher rates of depression, suicide, self-harm and eating disorders than their peers [97]. Similarly, patients with GD who are correctly framed and directed towards a multidisciplinary path for the resolution of their pathology have a clear reduction in psychiatric comorbidities. These data underline even more the importance of early and timely management in children and adolescents affected by GD [98, 99].

### The management of the child with gender dysphoria

In recent years, the approach to children with GD has changed, thanks also to the creation of specialized referral centers [100]. Certainly, one of the most important things in the management of these patients is the psychological support to reduce the suffering deriving from their condition.

In any case, the pediatrician should try not to direct the patient autonomously along this path, but should always refer to specialists in the sector to avoid incorrect therapeutic choices. What is required is an overall multidisciplinary evaluation and not a single consultation with the reference specialist which could lead to a loss of focus on the correct clinical management [95].

From a psychotherapeutic point of view, there are three different approaches to the clinical management of these children. The first of these, also defined as *persuasive*, seeks to reduce cross-gender behaviors by trying to convince the child that gender identity must correspond to biological sex. Clearly, this approach is now considered outdated and strongly criticized by the scientific community as it seeks to divert the child’s behavior in a coercive manner [101, 102]. The second approach can be defined as *watchful waiting* trying to positively support the children in their choices, without implementing behaviors aimed at modifying their cross-gender attitudes, knowing that it is possible that GD could be transitory [103].

The last approach involves an *early affirmation* of the child’s transgender identification. In this case, an attempt is made to encourage the child in gender transition earlier from a social point of view and without pharmacological prescriptions, a process considered reversible [104]. The problem with this approach is that by encouraging the child to assume behaviors belonging to another gender, he may forget what it was like to belong to the original gender. Moreover, it has been seen that these children rarely go back on their transition path because they are afraid of the possible negative reaction of their peers and the possible marginalization that would result [105]. It is not easy to define which is the best clinical approach for children, for this reason, the figure of the pediatrician is crucial because, knowing the patient, may direct her/him towards the most suitable path for her/him. It is certainly true that these patients should be also evaluated and followed by the specialists of the referral center, but the *reference* figure should remain their pediatrician that should carefully follow the patient on the territory by supporting both the child and the family in the therapeutic choices that can be undertaken. It will be also her/his responsibility to remain in contact with any specialists who will take care of the child.

**Fig. 1:**
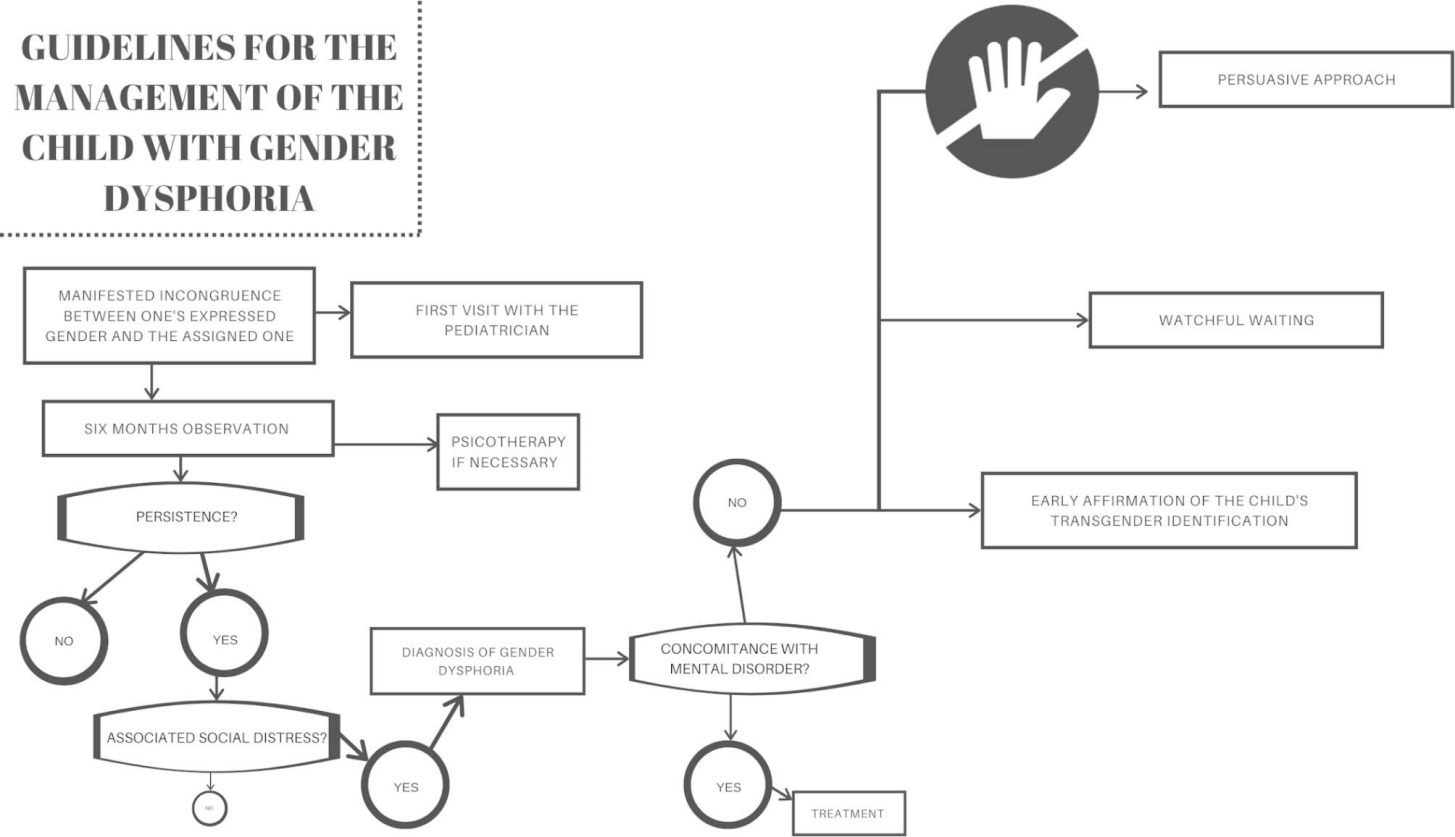
The flowchart explains the current guidelines for the clinical management of children with gender dysphoria

Figure 1 reassume the correct management of the children with GD.

### Management and treatment of the adolescents with gender dysphoria

Reviewing the literature about gender dysphoria in adolescents, what appears clear is that adolescence it’s not an easily definable time frame, because its onset can vary if we consider the physical progress of puberty or if we consider the age of onset of the same. Even so, it’s quite common for some pubertally advanced individuals not to be emotionally mature [106, 107]. Anyway, the physical changes typical of puberty and the first romantic or sexual experiences make adolescence a crucial period of gender identity development in gender non-conforming young people. The persistence rate of children diagnosed with gender dysphoria may vary, in natal males, it ranges from 2.2 to 30%, while in natal females, it from 12 to 50%, while most likely it may persist into adulthood when it is present during the adolescence [38]. It’s also possible for individuals to present gender dysphoria after the start of puberty: those patients, mostly natal boys, form a special population that usually goes through a more challenging treatment course, especially when they are not attracted to the natal sex [2, 87].

During adolescence, the clinical management of patients affected by gender dysphoria is inevitably more complicated since this is the period in which boys and girls have their first sexual experiences and develop their sexual identity. This is particularly true if we consider the increasing prevalence of the phenomenon found in some populations, which can be due to the surrounding society and extensive media coverage of gender non-conforming topics in the society nowadays, empowering a category of people who have been forced to hide their true self in the past decades [84]. As we mentioned before, it is pretty common for adolescents with gender dysphoria to engage in sexual intercourse with people forming part of their natal sex and, in doing so, they tend to hide their genitals. Therefore, it would be clinically useful for the pediatrician to ask about sexual activities when there are hints that the patient could be sexually active. It is necessary to do it in a safe environment without other people, such as parents or legal tutors, that could nullify the truthfulness of the inquiry [80]. A clinician should always keep in mind the existing difference between **gender identity** and **sexual orientation**, but it is doubtless that the majority of transgender people are attracted to their birth sex, and, in some socio-economically disadvantaged contexts, to the lack of existing or effective treatments, they are wrongly labeled as homosexuals [14]. Furthermore, trans-inclusive sexual education, which covers a specific topic that is necessary for gender non-conforming adolescents (i.e. contraception, sex acts aside from penile-vaginal sex, gender-affirming medical intervention), is effective in increasing condom and contraceptive use and reducing sexually transmitted diseases. [108, 109].

An interdisciplinary approach is preferred and has shown higher effectiveness [110]. The clinicians agree that a proper intervention should consider the psychological and clinical components. They suggest focusing on the social components, with attention to the school environment, and on the psychological aspect which must involve both the patient and her/his family. It is not uncommon for some parents to be emotionally unprepared for the complexity of the situation [10].

This multidisciplinary approach should include [46, 111]:

1. Endocrinological counseling, for the hormonal management;
2. Psychological support, for the psychopharmacology management;
3. Surgical counseling, to evaluate the possibility of surgery.

It’s worth mentioning the “six Cs” approach proposed by Ndoro consisting of care, compassion, competence, communication, courage and commitment, could provide high-quality care; consequently, the professionals involved in the clinical management of these patients should possess all these qualities [110].

Transitioning pathways go through some different phases, that can be summed up as follows [46, 112]:

- Evaluation by a multidisciplinary team from a nationally recognized reference center. Actually, in Italy there are eight referral centers for minors [46].
- Fertility counseling, to communicate to the patient the possibility of cryopreserving the semen or oocytes and the effects on fertility of hormone therapy.
- Hormone therapy consists of GnRH analogs or cross-sex steroids. This should be used for patients sixteen years old or older.
- The real-life test consists in evaluating how the patient is in society during his transition.
- A legal procedure for sex change.
- For surgical sex reassignment, it would be preferable to do it at the age of majority for legal implications after careful advice of the risk for the patient of this procedure.
- Social reintegration.
- Follow up.

It should be also noted that there are three different types of clinical interventions for these patients:

- **reversible**, which includes the use of GnRH analogues to delay the changes resulting from puberty. Other treatments such as progestogens or drugs such as spironolactone can be used to reduce the androgens produced by the testes. Oral contraceptives can be given to suppress menstruation.
- **partially reversible**, these interventions include hormonal therapies to masculinize or feminize the body. These treatments may require corrective surgery after a decision to interrupt the transitional process.
- **irreversible**, in the case of surgical procedures.

It is important to remember that irreversible treatment should be chosen only when the adolescent and his family have acknowledged the effects of the previous phases [113, 114].

Treatment with GnRH analogues inhibits the release of gonadotropins with a consequent blockade of puberty and has to be started at the Tanner stage 2 or 3 [114].

In the males these two stages can be summarized as follows [115]:

- **Tanner II**: testicular volume between 1.6 and 6 ml; skin on scrotum thins, reddens and enlarges; penis length unchanged, a small amount of long, downy hair with slight pigmentation at the base of the penis and scrotum.
- **Tanner III**: testicular volume between 6 and 12 ml; scrotum enlarges further; penis begins to lengthen, hair becomes more coarse and curly and begins to extend laterally.

In the females the two stages of Tanner can be described as follows [115]:

- **Tanner II**: breast bud forms, with a small area of surrounding glandular tissue; areola begins to enlarge, a small amount of long, downy hair with slight pigmentation on the major labia.
- **Tanner III**: breast begins to become more elevated, and extends beyond the borders of the areola, which continues to enlarge but remains in contour with the surrounding breast, hair becomes more coarse and curly, and begins to extend laterally.

The GnRH analogues are administered subcutaneously or intramuscularly quarterly [116]. The treatment stops menstruation and makes the mammary gland atrophic, while in males it blocks the growth of the beard. This therapy can lead to adverse effects including redness, sweating, headache and mood disturbances [112, 116]. It is crucial to underpin that the treatment alters fertility. Therefore, before starting therapy with GnRH analogues, it is necessary to carefully discuss with the patient to understand if she/he intends to keep the oocytes or spermatozoa for possible future reproduction. This is quite valid if the patient has reached at least the Tanner stage 3, on the contrary, if the patient is at Tanner stage 2 it will not be possible to cryopreserve the gametes. It must be said that these patients if they decide to interrupt the therapy with GnRH analogues, will take about six months to reactivate the hypothalamus-pituitary axis and will have a resumption of pubertal development [1, 116].

Subsequently, virilizing hormones (androgens) can be used in the female-to-male transition therapy and feminizing hormones (estrogen) for the male-to-female one; this should be done with gradually increasing dosages to assess the patient’s correct tolerance. Finally, it will be possible to choose to start the surgical procedure in agreement with the multidisciplinary team after a period defined as “social gender role transition”, which serves to assess how the subject lives in the society and gender in which she/he identifies [46].

For patients whose age ranges from 12 to 16, who are at Tanner stage 2 or 3 of puberty, according to the latest guidelines, it could be appropriate to start the hormonal treatment with GnRH analogues in the following cases:

- if the gender dysphoria was present also in childhood and it has persisted or exacerbated after the beginning of puberty.
- if it has been excluded that the patient suffers from psychiatric diseases or other clinical and social conditions that may interfere with the treatment.
- if there are social and psychological supports and awareness of the effects of the therapy [2, 113].

The benefit of such hormonal therapy is a better aesthetic result and the necessity of surgery in adulthood in these patients is reduced. These patients seem to have a lower level of anxiety and depression rates and a better social adjustment [104, 114]. The pediatrician should be aware that there are no consequences at the skeletal and metabolic levels, according to the literature [27, 46] About the timing of surgery, which has a satisfaction rate of the 87–97%, it could be useful to refer to Table 3, where it is shown how important it is to respect the patient’s wishes to avoid undesired treatments [46, 114, 117]. Ultimately, it may be worth mentioning the growing phenomenon of the so-called ‘regretters’, or of those who reject the transitional path undertaken although the percentage of people expressing no regret about transitioning is 98.4%. “Detransitioners” stress out the precocity of the treatment prescribed by the gender health providers through either puberty blockers and cross-sex hormones or gender reassignment surgery as opposed to the lack of psychological assistance. The high rate of suicide that can be found among this population probably shows that these people were affected by a mental disorder and that they were seeking medical intervention out of the hope that this would remove these psychological problems [97]. The disappointment usually led them to an escalation of self-harm and suicidal ideation, as resentment and hatred towards themselves [97]. It is therefore important that the professionals involved in the management of patients with gender dysphoria know how to guide them correctly in the transitional process considering the psychological difficulties that these patients often face [94, 109, 118, 119].

**Table 3:** The table shows the current therapeutic possibilities in adolescent patients with gender dysphoria

	_EVALUATION_ [2, 87]	_HORMONAL TREATMENT_ [104, 114, 116, 121]	SURGICAL TREATMENT [46, 114, 123]				
TIMING	6 MONTHS	12 MONTHS	2–4 weeks	4–6 weeks	3–6 months	6–12 months	
MALE TO FEMALE (MTF)	Gender Dysphoria Diagnosis	GnRH analogue/ Cross-sex steroids	Orchiectomy	Breast augmentation surgery	Feminizing genitoplasty surgery without vagina	Feminizing genitoplasty surgery with vagina	
FEMALE TO MALE (FTM)			Hysterectomy Annexectomy	Mastectomy	Metoidioplasty	Phalloplasty or penile prosthesis	

**Legend**:

**FTM =** gender transition of a transgender man who was assigned female at birth (AFAB)

**Hysterectomy** = surgical removal of the uterus and the cervix in most occasions

**Annexectomy** = excision of both ovaries and fallopian tubes

**Mastectomy** = bilateral surgical removing of the entire breast

**Metoidioplasty** = surgical procedure consisting in the cutting of ligaments around the erectile tissue of the clitoris to release it from the pubis and lengthen it to 4 or 6 cm with the possible incorporation of the urethra

**Phalloplasty** = surgery consisting in the construction of a penis with a penile prosthesis, in order for an erection to be achieved, and extension of the urethra to allow the patient to urinate standing

**MTF =** gender transition of a transgender woman who was assigned male at birth (AMAB)

**Orchiectomy** = surgical procedure consisting in the removal of both testicle

**Breast augmentation =** surgery technique involving breast implants

**Feminizing genitoplasty** = plastic surgery consisting in the construction of a vagina, by means of penile inversion or using sigmoid colon or scrotal tissue, clitoris and labia

The international medical community stresses the importance of the general practitioner’s involvement in the follow-up of a patient with gender dysphoria. This professional figure should be familiarized with the topic and help with the management of adults, for what concerns therapy or even the diagnosis. It shouldn’t come as a surprise that this topic can be even stronger for children and adolescents struggling with gender dysphoria, whose therapy and clinical managements in general need to be overseen by the primary care pediatrician. This undoubtedly would help achieve a better outcome on a long-term basis or an early recognition of signs and symptoms. Furthermore, the pediatric patients, since they possess different biological features in comparison with their adult counterparts, could benefit from their primary-care pediatrician advising the specialists who have to prescribe the therapy. Ultimately it should always be reminded that the transgender care elicits also some legal implications and informed consent for minors is a delicate issue especially when there is a conflicting relationship with the parents. Especially in those cases, the undisputed medical authority of the pediatrician may crucially support the achievement of the physical and psychological wellness of the adolescent/child [120–122].

**Fig. 2:**
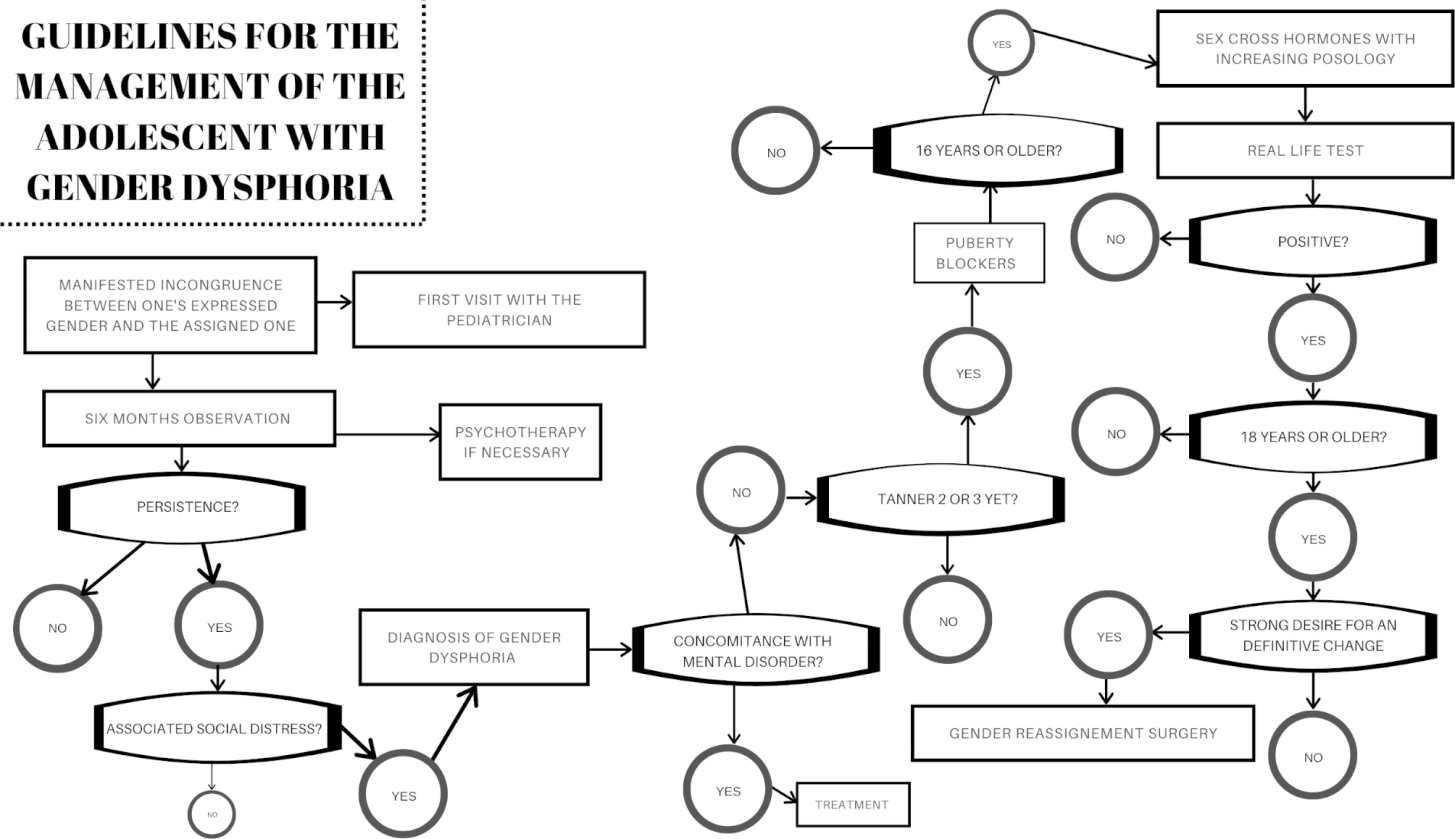
The flowchart explains the current guidelines for the clinical management of adolescents with gender dysphoria

Figure 2; Table 3 show the correct management of the adolescent with GD.

## Conclusions

Gender dysphoria is a clinical condition that is currently more frequent than in the past and therefore deserves correct knowledge by clinicians. The pediatrician, who is very often the first to interact with these patients, should be able to manage the patients, directing them towards a multidisciplinary path led by a team of experts in the field. For this reason, it is essential that the pediatrician is correctly supported by neuropsychiatric colleagues and specialized psychologists to formulate a correct diagnosis and refer the patient to the reference center. This aspect is fundamental since the pediatrician must not only offer his/her single point of view, but must integrate his/her experiences with those of dedicated specialists to guarantee a suitable therapeutic pathway for the patient.

It is therefore important that the pediatrician should be ready to subtle disclose cases of gender dysphoria. The pediatrician should use an inclusive and comprehensive language to finely support and comfort both the family and patient, unveiling the individual needs. Furthermore, the pediatrician should be aware of intermediate behaviors, such as gender variance, that usually tend to resolve with the growth and that will not necessarily involve the need to undertake a transitional path.

In conclusion, it is important that the pediatrician should be carefully informed on these issues, not thinking of them as sporadic to wisely coordinate the therapeutic paths of these patients according to the latest evidence.

## Electronic supplementary material

Below is the link to the electronic supplementary material.

Supplementary Material 1

## Data Availability

Not applicable.

## Abbreviations

AMAB: Assigned male at birth
AFAB: Assigned female at birth
GD: Gender dysphoria
GV: Gender variance
DSD: Disorders of sex development
DSM: Diagnostic and statistical manual of mental disorders
BDNF: Brain derived neurotrophic factor
TNF-a: Tumor necrosis factor-alpha
IL: Interleukin
GnRH: Gonadotrophin releasing hormone
FTM: Female to male
MTF: Male to female

## References

[1] Skordis N, Kyriakou A, Dror S, Mushailov A, Nicolaides NC. Gender dysphoria in children and adolescents: an overview. Hormones 2020;19. 10.1007/s42000-020-00174-1. 10.1007/s42000-020-00174-1 32020566

[2] American Psychiatric Association. Diagnostic and statistical Manual of Mental Disorders. American Psychiatric Association; 2013. 10.1176/appi.books.9780890425596.

[3] Calzo JP, Blashill AJ. Child sexual orientation and gender identity in the adolescent brain Cognitive Development Cohort Study. JAMA Pediatr. 2018;172. 10.1001/jamapediatrics.2018.2496. 10.1001/jamapediatrics.2018.2496 PMC6584307 30208469

[4] Schudson ZC, Beischel WJ, van Anders SM. Individual variation in Gender/Sex category definitions. Psychol Sex Orientat Gend Divers. 2019;6. 10.1037/sgd0000346.

[5] Griffiths DA. Shifting syndromes: sex chromosome variations and intersex classifications. Soc Stud Sci. 2018;48. 10.1177/0306312718757081. 10.1177/0306312718757081 PMC5808814 29424285

[6] Witchel SF. Disorders of sex development. Best Pract Res Clin Obstet Gynaecol. 2018;48. 10.1016/j.bpobgyn.2017.11.005. 10.1016/j.bpobgyn.2017.11.005 PMC5866176 29503125

[7] Dèttore D, Ristori J, Casale S. GID and gender-variant children in Italy: a study in preschool children. J Gay Lesbian Ment Health. 2011;15. 10.1080/19359705.2011.530569.

[8] Ristori J, Steensma TD (2016). Gender dysphoria in childhood. Int Rev Psychiatry.

[9] Smith K. Gender roles and development in Transgender Children. UC Merced Undergraduate Research Journal. 2015;8. 10.5070/m481029502.

[10] Butler C, Beavis J, Aldallal F, Nelson-Hall S, Shah-Beckley I (2021). GENDER VARIANCE: CHILDREN, ADOLESCENTS, PARENTS. J Fam Ther.

[11] Bailey JM, Vasey PL, Diamond LM, Breedlove SM, Vilain E, Epprecht M. Sexual orientation, controversy, and science. Psychol Sci Public Interest. 2016;17. 10.1177/1529100616637616. 10.1177/1529100616637616 27113562

[12] Shields JP, Cohen R, Glassman JR, Whitaker K, Franks H, Bertolini I. Estimating population size and demographic characteristics of lesbian, gay, bisexual, and transgender youth in middle school. J Adolesc Health 2013;52. 10.1016/j.jadohealth.2012.06.016. 10.1016/j.jadohealth.2012.06.016 23332492

[13] Katz-Wise SL, Hyde JS. Sexual fluidity and related attitudes and beliefs among young adults with a same-gender orientation. Arch Sex Behav. 2015;44. 10.1007/s10508-014-0420-1. 10.1007/s10508-014-0420-1 25378265

[14] Katz-Wise SL. Sexual fluidity in young adult women and men: associations with sexual orientation and sexual identity development. Psychol Sex. 2015;6. 10.1080/19419899.2013.876445.

[15] Gülgöz S, Glazier JJ, Enright EA, Alonso DJ, Durwood LJ, Fast AA, et al. Similarity in transgender and cisgender children’s gender development. Proc Natl Acad Sci U S A. 2019;116. 10.1073/pnas.1909367116. 10.1073/pnas.1909367116 PMC6900519 31740598

[16] Poirier F, Condat A, Laufer L, Rosenblum O, Cohen D. Non-binary gender and transgender youth: a literature review. Neuropsychiatr Enfance Adolesc. 2019;67. 10.1016/j.neurenf.2018.08.004.

[17] Chew D, Tollit MA, Poulakis Z, Zwickl S, Cheung AS, Pang KC. Youths with a non-binary gender identity: a review of their sociodemographic and clinical profile. Lancet Child Adolesc Health 2020;4. 10.1016/S2352-4642(19)30403-1. 10.1016/S2352-4642(19)30403-1 31978373

[18] Drescher J. Queer diagnoses parallels and contrasts in the history of homosexuality, gender variance, and the Diagnostic and Statistical Manual (DSM) Review and Recommendations prepared for the DSM-V sexual and gender identity Disorders Work Group. Focus (Madison). 2020;18. 10.1176/appi.focus.18302. 10.1176/appi.focus.18302 PMC7587913 33343243

[19] Deutsch MB. Making it count: improving estimates of the size of transgender and gender nonconforming populations. LGBT Health. 2016;3. 10.1089/lgbt.2016.0013. 10.1089/lgbt.2016.0013 27135657

[20] Richards C, Bouman WP, Seal L, Barker MJ, Nieder TO, Tsjoen G. Non-binary or genderqueer genders. Int Rev Psychiatry 2016;28. 10.3109/09540261.2015.1106446. 10.3109/09540261.2015.1106446 26753630

[21] Blechner MJ. Bigenderism and bisexuality. Contemp Psychoanal. 2015;51. 10.1080/00107530.2015.1060406.

[22] Davy Z, Toze M (2018). What is gender dysphoria? A critical systematic narrative review. Transgend Health.

[23] Battle DE. Diagnostic and statistical Manual of Mental Disorders (DSM). Volume 25. American Psychiatric Publishing, Inc; 2013. 10.4135/9781412956321.n79.

[24] Leibowitz S, De Vries ALC (2016). Gender dysphoria in adolescence. Int Rev PSYCHIATRY.

[25] Simons LK, Leibowitz SF, Hidalgo MA. Understanding gender variance in children and adolescents. Pediatr Ann. 2014;43. 10.3928/00904481-20140522-07. 10.3928/00904481-20140522-07 24972420

[26] Bonifacio HJ, Rosenthal SM. Gender variance and dysphoria in children and adolescents. Pediatr Clin North Am. 2015;62. 10.1016/j.pcl.2015.04.013. 10.1016/j.pcl.2015.04.013 26210629

[27] Puszczyk M, Czajeczny D. Gender dysphoria and gender variance in children – Diagnostic and therapeutic controversies. Archives of Psychiatry and Psychotherapy 2017;19. 10.12740/APP/74640.

[28] van der Steensma TD, Verhulst FC, Cohen-Kettenis PT. Gender variance in childhood and sexual orientation in adulthood: A prospective study. J Sex Med 2013;10. 10.1111/j.1743-6109.2012.02701.x. 10.1111/j.1743-6109.2012.02701.x 22458332

[29] Ristori J, Steensma TD. Gender dysphoria in childhood. Int Rev Psychiatry 2016;28. 10.3109/09540261.2015.1115754. 10.3109/09540261.2015.1115754 26754056

[30] Bower H. The gender identity disorder in the DSM-IV classification: a critical evaluation. Aust N Z J Psychiatry. 2001;35. 10.1046/j.1440-1614.2001.00859.x. 10.1046/j.1440-1614.2001.00859.x 11270443

[31] de Vries ALC, Beek TF, Dhondt K, de Vet HCW, Cohen-Kettenis PT, Steensma TD, et al. Reliability and clinical utility of gender identity-related Diagnoses: comparisons between the ICD-11, ICD-10, DSM-IV, and DSM-5. LGBT Health. 2021;8. 10.1089/lgbt.2020.0272. 10.1089/lgbt.2020.0272 33600259

[32] Drescher J, Cohen-Kettenis PT, Reed GM (2016). Gender incongruence of childhood in the ICD-11: controversies, proposal, and rationale. Lancet Psychiatry.

[33] De Vries ALC, Beek TF, Dhondt K, De Vet HCW, Cohen-Kettenis PT, Steensma TD (2021). Reliability and clinical utility of gender identity-related Diagnoses: comparisons between the ICD-11, ICD-10, DSM-IV, and DSM-5. LGBT Health.

[34] Drescher J (2014). Controversies in gender diagnoses. LGBT Health.

[35] Falletti E (2018). Protecting children affected by atypical gender Identity Organization: the comparative legal perspective. SSRN Electron J.

[36] Winter S, De Cuypere G, Green J, Kane R, Knudson G. The proposed ICD-11 gender incongruence of Childhood diagnosis: a World Professional Association for Transgender Health Membership Survey. Arch Sex Behav. 2016;45. 10.1007/s10508-016-0811-6. 10.1007/s10508-016-0811-6 27492343

[37] Weiselberg EC, Shadianloo S. Overview of care for transgender children and youth. Curr Probl Pediatr Adolesc Health Care. 2019;49. 10.1016/j.cppeds.2019.100682. 10.1016/j.cppeds.2019.100682 31706835

[38] Zucker KJ. Epidemiology of gender dysphoria and transgender identity. Sex Health. 2017;14. 10.1071/SH17067. 10.1071/SH17067 28838353

[39] Clark TC, Lucassen MFG, Bullen P, Denny SJ, Fleming TM, Robinson EM et al. The health and well-being of transgender high school students: Results from the New Zealand adolescent health survey (youth’12). J Adolesc Health 2014;55. 10.1016/j.jadohealth.2013.11.008. 10.1016/j.jadohealth.2013.11.008 24438852

[40] Eisenberg ME, Gower AL, McMorris BJ, Rider GN, Shea G, Coleman E. Risk and Protective Factors in the Lives of Transgender/Gender Nonconforming Adolescents. J Adolesc Health 2017;61. 10.1016/j.jadohealth.2017.04.014. 10.1016/j.jadohealth.2017.04.014 PMC5626022 28736148

[41] Cohen-Kettenis PT, Owen A, Kaijser VG, Bradley SJ, Zucker KJ. Demographic characteristics, social competence, and behavior problems in children with gender identity disorder: a cross-national, cross-clinic comparative analysis. J Abnorm Child Psychol. 2003;31. 10.1023/A:1021769215342. 10.1023/a:1021769215342 12597698

[42] Wood H, Sasaki S, Bradley SJ, Singh D, Fantus S, Owen-Anderson A, et al. Patterns of referral to a gender identity service for children and adolescents (1976–2011): age, sex ratio, and sexual orientation. J Sex Marital Ther. 2013;39. 10.1080/0092623X.2012.675022. 10.1080/0092623X.2012.675022 23152965

[43] Zucker KJ, Lawrence AA. Epidemiology of gender identity disorder: recommendations for the standards of care of the world professional association for transgender health. Int J Transgenderism. 2009;11. 10.1080/15532730902799946.

[44] Reisner SL, Conron KJ, Scout S, Baker K, Herman JL, Lombardi E, et al. Counting” transgender and gender-nonconforming adults in Health Research: recommendations from the gender identity in US Surveillance Group. TSQ: Transgender Studies Quarterly. 2015;2. 10.1215/23289252-2848877.

[45] Sabina Pignataro. Bambini e adolescenti transgender, i numeri veri e le parole giuste. VITA Magazine; 2019.

[46] http://www.onig.it/drupal8/docs/SoC_minorenni. ONIG linee guida per la presa in carico dei minorenni con sviluppo atipico dell’identità di genere. n.d.

[47] Fisher AD, Ristori J, Morelli G, Maggi M. The molecular mechanisms of sexual orientation and gender identity. Mol Cell Endocrinol. 2018;467. 10.1016/j.mce.2017.08.008. 10.1016/j.mce.2017.08.008 28847741

[48] Ristori J, Cocchetti C, Romani A, Mazzoli F, Vignozzi L, Maggi M, et al. Brain sex differences related to gender identity development: genes or hormones? Int J Mol Sci. 2020;21. 10.3390/ijms21062123. 10.3390/ijms21062123 PMC7139786 32204531

[49] Bentz EK, Schneeberger C, Hefler LA, van Trotsenburg M, Kaufmann U, Huber JC, et al. A common polymorphism of the SRD5A2 gene and transsexualism. Reproductive Sci. 2007;14. 10.1177/1933719107306230. 10.1177/1933719107306230 18000232

[50] Ercan O, Kutlug S, Uysal O, Alikasifoglu M, Inceoglu D. Gender identity and gender role in DSD patients raised as females: a preliminary outcome study. Front Endocrinol (Lausanne). 2013;4. 10.3389/fendo.2013.00086. 10.3389/fendo.2013.00086 PMC3711069 23874323

[51] Cohen-Kettenis PT. Gender change in 46,XY persons with 5α-reductase-2 deficiency and 17β-hydroxysteroid dehydrogenase-3 deficiency. Arch Sex Behav. 2005;34. 10.1007/s10508-005-4339-4. 10.1007/s10508-005-4339-4 16010463

[52] Fisher AD, Castellini G, Casale H, Fanni E, Bandini E, Campone B, et al. Hypersexuality, paraphilic behaviors, and gender dysphoria in individuals with Klinefelter’s syndrome. J Sex Med. 2015;12. 10.1111/jsm.13048. 10.1111/jsm.13048 26612786

[53] Fiore M, Tarani L, Radicioni A, Spaziani M, Ferraguti G, Putotto C, et al. Serum prokineticin-2 in prepubertal and adult Klinefelter individuals. Can J Physiol Pharmacol. 2021;100. 10.1139/cjpp-2021-0457. 10.1139/cjpp-2021-0457 34614364

[54] Erişen Karaca S, Eröz R, Arslanoğlu İ. A rare cause of female gender dysphoria: report of three cases with low percentage of turner mosaicism. Duzce Med J. 2020;22. 10.18678/dtfd.780970.

[55] Tarani L, Ceci FM, Carito V, Ferraguti G, Petrella C, Greco A, et al. Neuroimmune Dysregulation in Prepubertal and adolescent individuals affected by Klinefelter Syndrome. Endocr Metab Immune Disord Drug Targets. 2022;22. 10.2174/1871530322666220704101310. 10.2174/1871530322666220704101310 35794745

[56] Herrmann L, Bindt C, Schweizer K, Micheel J, Nieder TO, Haa J, et al. Autism Spectrum Disorders and gender dysphoria among children and adolescents: systematic review on the Co-Occurrence. Psychiatr Prax. 2020;47. 10.1055/a-1148-4873. 10.1055/a-1148-4873 32542639

[57] n der Miesen AIR, Hurley H, de Vries ALC. Gender dysphoria and autism spectrum disorder: A narrative review. Int Rev Psychiatry 2016;28. 10.3109/09540261.2015.1111199. 10.3109/09540261.2015.1111199 26753812

[58] Tarani L, Carito V, Ferraguti G, Petrella C, Greco A, Ralli M et al. Neuroinflammatory Markers in the Serum of Prepubertal Children with down Syndrome. J Immunol Res 2020;2020. 10.1155/2020/6937154. 10.1155/2020/6937154 PMC7125499 32280719

[59] Petrella C, Spaziani M, D’Orazi V, Tarani L, Terracina S, Tarani F et al. Prokineticin 2/PROK2 and Male Infertility. Biomedicines 2022;10. 10.3390/biomedicines10102389. 10.3390/biomedicines10102389 PMC9598863 36289651

[60] Zubiaurre-Elorza L, Junque C, Gómez-Gil E, Guillamon A. Effects of cross-sex hormone treatment on cortical thickness in transsexual individuals. J Sex Med 2014;11. 10.1111/jsm.12491. 10.1111/jsm.12491 24617977

[61] Manzouri A, Savic I. Possible neurobiological underpinnings of homosexuality and gender dysphoria. Cereb Cortex. 2019;29. 10.1093/cercor/bhy090. 10.1093/cercor/bhy090 PMC6677918 30084980

[62] Hahn A, Kranz GS, Küblböck M, Kaufmann U, Ganger S, Hummer A, et al. Structural connectivity networks of transgender people. Cereb Cortex. 2015;25. 10.1093/cercor/bhu194. 10.1093/cercor/bhu194 PMC4585501 25217469

[63] Pluchino N, Russo M, Santoro AN, Litta P, Cela V, Genazzani AR. Steroid hormones and BDNF. Neuroscience 2013;239. 10.1016/j.neuroscience.2013.01.025. 10.1016/j.neuroscience.2013.01.025 23380505

[64] Fiore M, Triaca V, Amendola T, Tirassa P, Aloe L (2002). Brain NGF and EGF administration improves passive avoidance response and stimulates brain precursor cells in aged male mice. Physiol Behav.

[65] Ferraguti G, Terracina S, Micangeli G, Lucarelli M, Tarani L, Ceccanti M (2023). NGF and BDNF in pediatrics syndromes. Neurosci Biobehav Rev.

[66] Ferraguti G, Fanfarillo F, Tarani L, Blaconà G, Tarani F, Barbato C (2022). NGF and the male Reproductive System: potential clinical applications in infertility. Int J Mol Sci.

[67] Petrella C, Nenna R, Petrarca L, Tarani F, Paparella R, Mancino E (2022). Serum NGF and BDNF in Long-COVID-19 adolescents: a pilot study. Diagnostics.

[68] Tore F, Tonchev A, Fiore M, Tuncel N, Atanassova P, Aloe L (2007). From adipose tissue protein secretion to Adipopharmacology of Disease. Immunol Endocr Metab Agents Med Chem.

[69] van den Notaras M. Neurobiology of BDNF in fear memory, sensitivity to stress, and stress-related disorders. Mol Psychiatry. 2020;25. 10.1038/s41380-019-0639-2. 10.1038/s41380-019-0639-2 31900428

[70] Angelucci F, Piermaria J, Gelfo F, Shofany J, Tramontano M, Fiore M (2016). The effects of motor rehabilitation training on clinical symptoms and serum BDNF levels in Parkinson’s disease subjects. Can J Physiol Pharmacol.

[71] Fuss J, Biedermann S, Stalla GK, Auer MK. On the quest for a biomechanism of transsexualism: is there a role for BDNF? J Psychiatr Res. 2013;47. 10.1016/j.jpsychires.2013.08.023. 10.1016/j.jpsychires.2013.08.023 24070909

[72] Schneider MA, Andreazza T, Fontanari AMv, Costa AB, da Silva DC, de Aguiar BW, et al. Serum concentrations of brain-derived neurotrophic factor in patients diagnosed with gender dysphoria undergoing sex reassignment surgery. Trends Psychiatry Psychother. 2017;39. 10.1590/2237-6089-2016-0033. 10.1590/2237-6089-2016-0033 28403322

[73] Real AG, Fontanari AMV, Costa AB, Soll BMB, Bristot G, de Oliveira LF (2021). Gender dysphoria: prejudice from childhood to adulthood, but no impact on inflammation. A cross-sectional controlled study. Trends Psychiatry Psychother.

[74] Micangeli G, Menghi M, Profeta G, Tarani F, Mariani A, Petrella C et al. The Impact of Oxidative Stress on Pediatrics Syndromes. Antioxidants 2022;11:1983. 10.3390/antiox11101983. 10.3390/antiox11101983 PMC9598789 36290706

[75] Aloe L, Fiore M (1997). TNF-α expressed in the brain of transgenic mice lowers central tyroxine hydroxylase immunoreactivity and alters grooming behavior. Neurosci Lett.

[76] Tarani L, Carito V, Ferraguti G, Petrella C, Greco A, Ralli M (2020). Neuroinflammatory markers in the serum of Prepubertal Children with down Syndrome. J Immunol Res.

[77] Fiore M, Petrella C, Coriale G, Rosso P, Fico E, Ralli M (2022). Markers of Neuroinflammation in the serum of Prepubertal Children with fetal Alcohol Spectrum Disorders. CNS Neurol Disord Drug Targets.

[78] Costa AB, Fontanari AMv, Andreazza T, Salvador J, Koff WJ, Aguiar B, et al. BDNF: a biomarker for social vulnerability in individuals diagnosed with gender dysphoria. J Psychiatr Res. 2014;50. 10.1016/j.jpsychires.2013.11.009. 10.1016/j.jpsychires.2013.11.009 24332481

[79] Tornese G, di Grazia M, Roia A, Morini G, Cosentini D, Carrozzi M et al. Disforia di genere e dintorni. Medico e Bambino 2016;35.

[80] de Vries ALC, Klink D, Cohen-Kettenis PT. What the Primary Care Pediatrician Needs to Know About Gender Incongruence and Gender Dysphoria in Children and Adolescents. Pediatr Clin North Am 2016;63. 10.1016/j.pcl.2016.07.011. 10.1016/j.pcl.2016.07.011 27865337

[81] Tarani L, Rasio D, Tarani F, Parlapiano G, Valentini D, Dylag KA, et al. Pediatrics of disability: a comprehensive approach to the child with syndromic psychomotor delay. Curr Pediatr Rev. 2021;17. 10.2174/1573396317666211129093426. 10.2174/1573396317666211129093426 34844545

[82] Profeta G, Micangeli G, Tarani F, Paparella R, Ferraguti G, Spaziani M (2022). Sexual Developmental Disorders in Pediatrics. Clin Ter.

[83] Cretella M. Gender Dysphoria in Children American College of Pediatricians - June 2017. Issues Law Med 2017;32. 29108153

[84] Ristori J, Steensma TD. International Review of Psychiatry Gender dysphoria in childhood Gender dysphoria in childhood. Int Rev Psychiatry 2016;28. 10.3109/09540261.2015.1115754 26754056

[85] American Psychiatric Association. DSM-5 Diagnostic Classification. Diagnostic and Statistical Manual of Mental Disorders., 2013. 10.1176/appi.books.9780890425596.x00diagnosticclassification.

[86] Wylie K, Knudson G, Khan SI, Bonierbale M, Watanyusakul S, Baral S. Serving transgender people: clinical care considerations and service delivery models in transgender health. The Lancet 2016;388. 10.1016/S0140-6736(16)00682-6. 10.1016/S0140-6736(16)00682-6 27323926

[87] Byne W, Bradley SJ, Coleman E, Eyler AE, Green R, Menvielle EJ, et al. Report of the american psychiatric association task force on treatment of gender identity disorder. Arch Sex Behav. 2012;41. 10.1007/s10508-012-9975-x. 10.1007/s10508-012-9975-x 22736225

[88] Littman L, Correction. Parent reports of adolescents and young adults perceived to show signs of a rapid onset of gender dysphoria. PLoS ONE. 2019;14. 10.1371/journal.pone.0214157. 10.1371/journal.pone.0214157 PMC6424391 30889220

[89] Lopez X, Stewart S, Jacobson-Dickman E. Approach to children and adolescents with gender dysphoria. Pediatr Rev. 2016;37. 10.1542/pir.2015-0032. 10.1542/pir.2015-0032 26933223

[90] Tarani L, Micangeli G, Rasio D, Ottombrino S, Liberati N, de Angelis D, et al. Clinical and genetic approach to the dysmorphic child. Biomedical Reviews. 2018;29. 10.14748/bmr.v29.5848.

[91] de Vries ALC, Klink D, Cohen-Kettenis PT (2016). What the primary care pediatrician needs to know about gender incongruence and gender dysphoria in children and adolescents. Pediatr Clin North Am.

[92] Hermanutz K. Mental Health Issues in Transgender Children and Children with Gender Dysphoria. Canadian Journal of Family and Youth / Le Journal Canadien de Famille et de La Jeunesse 2021;13. 10.29173/cjfy29707.

[93] Wallien MSC, Cohen-Kettenis PT. Psychosexual outcome of gender-dysphoric children. J Am Acad Child Adolesc Psychiatry. 2008;47. 10.1097/CHI.0b013e31818956b9. 10.1097/CHI.0b013e31818956b9 18981931

[94] Steensma TD, Biemond R, de Boer F, Cohen-Kettenis PT. Desisting and persisting gender dysphoria after childhood: a qualitative follow-up study. Clin Child Psychol Psychiatry. 2011;16. 10.1177/1359104510378303. 10.1177/1359104510378303 21216800

[95] Weiselberg EC, Shadianloo S (2019). Overview of care for transgender children and youth. Curr Probl Pediatr Adolesc Health Care.

[96] Haley SG, Tordoff DM, Kantor AZ, Crouch JM, Ahrens KR (2019). Sex Education for Transgender and Non-Binary Youth: previous experiences and recommended content. J Sex Med.

[97] Frew T, Watsford C, Walker I. Gender dysphoria and psychiatric comorbidities in childhood: a systematic review. Aust J Psychol. 2021;73. 10.1080/00049530.2021.1900747.

[98] Price-Feeney M, Green AE, Dorison S. Understanding the Mental Health of Transgender and Nonbinary Youth. J Adolesc Health 2020;66. 10.1016/j.jadohealth.2019.11.314. 10.1016/j.jadohealth.2019.11.314 31992489

[99] Connolly MD, Zervos MJ, Barone CJ, Johnson CC, Joseph CLM. The Mental Health of Transgender Youth: Advances in Understanding. J Adolesc Health 2016;59. 10.1016/j.jadohealth.2016.06.012. 10.1016/j.jadohealth.2016.06.012 27544457

[100] di Ceglie D. Care for Gender-Dysphoric Children, 2014. 10.1007/978-1-4614-7441-8_8.

[101] Khatchadourian K, Amed S, Metzger DL. Clinical management of youth with gender dysphoria in vancouver. J Pediatr. 2014;164. 10.1016/j.jpeds.2013.10.068. 10.1016/j.jpeds.2013.10.068 24315505

[102] Hsieh S, Leininger J. Resource list: clinical care programs for gender-nonconforming children and adolescents. Pediatr Ann. 2014;43. 10.3928/00904481-20140522-11. 10.3928/00904481-20140522-11 24972419

[103] Giordano S. Children with Gender Identity Disorder: A Clinical, Ethical, and Legal Analysis. 2012. 10.4324/9780203097892.

[104] Baetens L, Dhondt K. Psychosocial challenges and hormonal treatment in gender diverse children and adolescents. A narrative review. Int J Impot Res. 2021;33. 10.1038/s41443-020-0291-z. 10.1038/s41443-020-0291-z 32366985

[105] Fuss J, Auer MK, Briken P. Gender dysphoria in children and adolescents: a review of recent research. Curr Opin Psychiatry. 2015;28. 10.1097/YCO.0000000000000203. 10.1097/YCO.0000000000000203 26382161

[106] Kaltiala-Heino R, Työläjärvi M, Lindberg N. Gender dysphoria in adolescent population: a 5-year replication study. Clin Child Psychol Psychiatry. 2019;24. 10.1177/1359104519838593. 10.1177/1359104519838593 30968719

[107] Safitri KH, Mukaramah S (2021). Gender Dysphoria in Adolescence. Asian Community Health Nursing Research.

[108] Selveindran NM, Syed Zakaria SZ, Jalaludin MY, Rasat R. Quality of Life in Children with Disorders of Sex Development. Horm Res Paediatr. 2017;88. 10.1159/000478780. 10.1159/000478780 28965114

[109] Haley SG, Tordoff DM, Kantor AZ, Crouch JM, Ahrens KR. Sex Education for Transgender and Non-Binary Youth: Previous Experiences and Recommended Content. J Sex Med 2019;16. 10.1016/j.jsxm.2019.08.009. 10.1016/j.jsxm.2019.08.009 31585806

[110] Ndoro S. Effective multidisciplinary working: the key to high-quality care. Br J Nurs. 2014;23. 10.12968/bjon.2014.23.13.724. 10.12968/bjon.2014.23.13.724 25072333

[111] Paparella R, Menghi M, Micangeli G, Leonardi L, Profeta G, Tarani F, et al. Autoimmune polyendocrine syndromes in the Pediatric Age. Child (Basel). 2023;10. 10.3390/children10030588. 10.3390/children10030588 PMC10047132 36980146

[112] Delemarre-Van De Waal HA, Cohen-Kettenis PT. Clinical management of gender identity disorder in adolescents: a protocol on psychological and paediatric endocrinology aspects. Eur J Endocrinol Supplement. 2006;155. 10.1530/eje.1.02231.

[113] Priest M. Transgender Children and the Right to Transition: Medical Ethics When Parents Mean Well but Cause Harm. Am J Bioeth 2019;19. 10.1080/15265161.2018.1557276. 10.1080/15265161.2018.1557276 30784385

[114] Mahfouda S, Moore JK, Siafarikas A, Hewitt T, Ganti U, Lin A et al. Gender-affirming hormones and surgery in transgender children and adolescents. Lancet Diabetes Endocrinol 2019;7. 10.1016/S2213-8587(18)30305-X. 10.1016/S2213-8587(18)30305-X 30528161

[115] Wood CL, Lane LC, Cheetham T, Puberty. Normal physiology (brief overview). Best Pract Res Clin Endocrinol Metab. 2019;33. 10.1016/j.beem.2019.03.001. 10.1016/j.beem.2019.03.001 31000487

[116] Martinerie L, Condat A, Bargiacchi A, Bremont-Weill C, de Vries MC, Hannema SE. Management of endocrine disease: Approach to the management of children and adolescents with gender dysphoria. Eur J Endocrinol. 2018;179. 10.1530/EJE-18-0227. 10.1530/EJE-18-0227 30049812

[117] de Vries ALC, McGuire JK, Steensma TD, Wagenaar ECF, Doreleijers TAH, Cohen-Kettenis PT. Young adult psychological outcome after puberty suppression and gender reassignment. Pediatrics 2014;134. 10.1542/peds.2013-2958. 10.1542/peds.2013-2958 25201798

[118] Plöderl M, Fartacek R. Childhood gender nonconformity and harassment as predictors of suicidality among gay, lesbian, bisexual, and heterosexual Austrians. Arch Sex Behav. 2009;38. 10.1007/s10508-007-9244-6. 10.1007/s10508-007-9244-6 18040769

[119] Evans M. Freedom to think: the need for thorough assessment and treatment of gender dysphoric children. BJPsych Bull. 2021;45. 10.1192/bjb.2020.72. 10.1192/bjb.2020.72 PMC8596151 32690120

[120] Parkinson P. Adolescent Gender Dysphoria and the Informed Consent Model of Care. J Law Med 2021;28. 34369127

[121] Hembree WC, Cohen-Kettenis PT, Gooren L, Hannema SE, Meyer WJ, Murad MH, et al. Endocrine treatment of gender-dysphoric/ gender-incongruent persons: an endocrine society∗clinical practice guideline. J Clin Endocrinol Metab. 2017;102. 10.1210/jc.2017-01658. 10.1210/jc.2017-01658 28945902

[122] Joseph A, Cliffe C, Hillyard M, Majeed A. Gender identity and the management of the transgender patient: a guide for non-specialists. J R Soc Med. 2017;110. 10.1177/0141076817696054. 10.1177/0141076817696054 PMC5407520 28382847

[123] Smith YLS, van Goozen SHM, Kuiper AJ, Cohen-Kettenis PT. Sex reassignment: outcomes and predictors of treatment for adolescent and adult transsexuals. Psychol Med. 2005;35. 10.1017/S0033291704002776. 10.1017/s0033291704002776 15842032

