# Supplementary material for: The role of the pediatrician in the management of the child and adolescent with gender dysphoria
Source: Ital J Pediatr. 2023 Jun 14;49:71. doi: 10.1186/s13052-023-01466-z (PMC10268477; doi:10.1186/s13052-023-01466-z)
Supplement: Supplementary file 1 — Supplementary Material 1 [file 13052_2023_1466_MOESM1_ESM.doc]

**Italian Journal of Pediatrics**

**Editor-in-Chief**

**Giovanni Corsello,**

**University of Palermo, Palermo, Italy**

Rome, Italy, April 16th, 2023

Subject: revised manuscript submission - ITJP-D-22-00262R2

Dear Professor Corsello

Please, find enclosed the revised report “**The Role of the Pediatrician in the Management of the Child and Adolescent with Gender Dysphoria**” by Ginevra Micangeli and co-workers that we would like to publish in “***Italian Journal of Pediatrics”.*** As you will see in the enclosed pages, we carefully took into account all the criticisms raised by the reviewers replying point-by-point to the comments. We highlighted the revised text of the paper.

Sincerely

Luigi Tarani

Marco Fiore

Prof. Luigi Tarani

Department of Pediatrics,

"Sapienza" University of Rome, Italy

Viale del Policlinico, 155

00161 Rome, Italy

[luigi.tarani@uniroma1.it](mailto:luigi.tarani@uniroma1.it)

*Editorial Board Italian Journal Pediatrics*

Dr. Marco Fiore Ph.D.

Department of Sense Organs

Sapienza University of Rome, Italy

Institute of Biochemistry and Cellular Biology. IBBC-CNR

Section of Neurobiology

Viale del Policlinico, 155 – 00161 Rome, Italy

marco.fiore@uniroma1.it

marco.fiore@cnr.it

Reviewer #1: thank you to the authors

All my requests were satisfied

*Reply: we do thank the reviewer*

Reviewer #2: the paper deserves to be published

*Reply: we do thank the reviewer*

Reviewer #3: Very interesting, exhaustive and well written paper.

*Reply: we do thank the reviewer for the positive comments*

I've noticed that at page 11 line 44 you have written "boy" but I think that you meant "boy/girl" or generically "child".

*Reply: as requested, appropriate modifications were done*
